# Adverse Drug Reactions with Drugs Used in Multiple Sclerosis: An Analysis from the Italian Pharmacovigilance Database

**DOI:** 10.3389/fphar.2022.808370

**Published:** 2022-02-23

**Authors:** Maria Antonietta Barbieri, Emanuela Elisa Sorbara, Alessandro Battaglia, Giuseppe Cicala, Vincenzo Rizzo, Edoardo Spina, Paola Maria Cutroneo

**Affiliations:** ^1^ Department of Clinical and Experimental Medicine, University of Messina, Messina, Italy; ^2^ Sicilian Regional Pharmacovigilance Centre, University Hospital of Messina, Messina, Italy

**Keywords:** multiple sclerosis, disease modifying therapies, injectable drugs, adverse drug reactions, pharmacovigilance

## Abstract

Given the importance of inflammation at the onset of multiple sclerosis (MS), therapy is mainly based on the use of anti-inflammatory drugs including disease modifying therapies (DMTs). Considering the recent approval of some DMTs, pharmacovigilance becomes a fundamental tool for the acquisition of new safety data. The aim of the study was to analyze adverse drug reactions (ADRs) related to the use of drugs approved for MS. All national publicly-available aggregated ADR reports recorded from 2002 to 2020 into the Reports of Adverse Reactions of Medicines (RAM) system and all complete Sicilian data reported into the Italian spontaneous reporting system (SRS) database having as suspected drugs interferon β-1a (IFN β-1a), interferon β-1b (IFN β-1b), peginterferon β-1a (PEG-IFN β-1a), glatiramer acetate (GA), natalizumab (NTZ), fingolimod (FNG), teriflunomide (TRF), dimethyl fumarate (DMF), alemtuzumab (Alem), ocrelizumab (OCZ), or cladribine (Cladr), were collected. Descriptive analyses of national, publicly-available aggregated data and full-access regional data were performed to assess demographic characteristics and drug-related variables followed by a more in-depth analysis of all Sicilian ADRs with a case-by-case assessment and a disproportionality analysis of unexpected ADRs. A total of 13,880 national reports have been collected from 2002 to 2020: they were mainly not serious ADRs (67.9% vs. 26.1%) and related to females (71.7% *vs.* 26.3%) in the age group 18–65 years (76.5%). The most reported ADRs were general and administration site conditions (*n* = 6,565; 47.3%), followed by nervous (*n* = 3,090; 22.3%), skin (*n* = 2,763; 19.9%) and blood disorders (*n* = 2,180; 15.7%). Some unexpected Sicilian ADRs were shown, including dyslipidemia for FNG (*n* = 10; ROR 28.5, CI 14.3–59.6), NTZ (*n* = 5; 10.3, 4.1–25.8), and IFN β-1a (*n* = 4; 8.7, 3.1–24.1), abortion and alopecia for NTZ (*n* = 9; 208.1, 73.4–590.1; *n* = 3; 4.9, 1.5–15.7), and vitamin D deficiency for GA (*n* = 3; 121.2, 30.9–475.3). Moreover, breast cancer with DMF (*n* = 4, 62.8, 20.5–191.9) and hypothyroidism with Cladr (*n* = 3; 89.2, 25.9–307.5) were also unexpected. The reporting of drugs-related ADRs in MS were mostly reported in the literature, but some unknown ADRs were also found. However, further studies are necessary to increase the awareness about the safety profiles of new drugs on the market.

## Introduction

Multiple sclerosis (MS) is chronic inflammatory disease with demyelination and axonal damage on the central nervous system (CNS), and autoimmune disease with a multifactorial etiology (Yamout and Alroughani, 2018). The disease is estimated to affect approximately 2.8 million people worldwide and it is the first cause of non-traumatic neurological disability in young people (The Multiple Sclerosis International Federation (MSIF), 2020; Yamout and Alroughani, 2018). Its prevalence is higher in females, especially in the age group 20–40 years (Filippi et al., 2018; Trojano et al., 2019). The autoimmune response of MS is initially mediated by T lymphocytes reactive to myelin proteins with a consequent infiltration of CD4+ T cells in the “acute demyelination plaques” (Compston and Coles, 2008; Filippi et al., 2018; Patsopoulos et al., 2019). At a later stage, B cells produce autoantibodies located in both oligoclonal bands and plaques (Arrambide et al., 2018). Moreover, monocytes and macrophages, as well as dendritic cells, play a key role in the immunopathogenesis of MS (Nuyts et al., 2013; Ma et al., 2019).

Given the importance of the inflammation at the onset of MS, the therapy is mainly based on the use of anti-inflammatory drugs. The introduction of disease modifying therapies (DMTs) has radically changed the treatment of MS for their ability to prevent and/or reduce the frequency of relapses as well as delay the progression of the disease (Giovannoni, 2018; Tintore et al., 2019). The first therapeutic strategy is characterized by immunostimulants including interferon β-1a (IFN β-1a), interferon β-1b (IFN β-1b), peginterferon β-1a (PEG-IFN β-1a), and glatiramer acetate (GA), followed by the recent introduction of immunosuppressants such as natalizumab (NTZ), fingolimod (FNG), teriflunomide (TRF), dimethyl fumarate (DMF), alemtuzumab (Alem), ocrelizumab (OCZ), and cladribine (Cladr). Of the First-line drugs, including IFNs and GA, are mostly associated with injection site reactions and flu-like symptoms (La Mantia et al., 2016). Of the new first-line oral therapies, DMF can cause flushing, gastrointestinal disturbances, and lymphopenia and TRF can cause diarrhea, nausea, headache, hepatotoxicity, alopecia, and elevated blood pressure (Pardo and Jones, 2017). All other previously cited drugs are used as second-line therapy and are characterized by a higher effectiveness, but an increased risk of adverse drug reactions (ADRs) (Gitto, 2017; Pardo and Jones, 2017). Moreover, FNG and Alem are related to cardiotoxicity (Faissner and Gold, 2018; Ahrabian et al., 2020), while NTZ to progressive multifocal leukoencephalopathy (PML) (Vermeer et al., 2015; Clerico et al., 2017; Butzkueven et al., 2020). Cladr appears to be better tolerated with mild to moderate ADRs predominantly concerning lymphopenia (Guarnera et al., 2017; Jacobs et al., 2018; Scott, 2019; Leist et al., 2020). Furthermore, pre-marketing studies showed an increased risk of neoplasms only with FNG (Guarnera et al., 2017; Lebrun and Rocher, 2018; Alping et al., 2020).

Considering that several DMTs have been recently approved, pharmacovigilance is a fundamental tool for the acquisition of new safety data to improve knowledge of the risk/benefit ratio of these drugs. In the last few years, several safety alerts on possible risks associated with DMTs have been issued by regulatory agencies, such as the onset of PML with Cladr (European Medicines Agency, 2017); the onset of basal cell cancer, lymphoma, and PML with FNG (European Medicines Agency, 2015); the contraindication of FNG in pregnancy due to the risk of congenital malformations (European Medicines Agency, 2019a); restrictions on the use of Alem for rare, but serious, side effects including fatal cases related to myocardial ischemia, myocardial infarction, cerebral hemorrhage, dissection of the cervico-cephalic arteries, pulmonary alveolar hemorrhage, and thrombocytopenia (European Medicines Agency, 2019b). The main objective of this study was to evaluate the characteristics of ADRs with drugs approved for MS through 1) the analysis of national open data from Reports of Adverse Reactions of Medicines (report Reazioni Avverse dei Medicinali, RAM) giving the global evaluation of ADRs in Italy, and 2) focusing on complete data from the regional Sicilian database allowing case-by-case causality assessment of ADRs followed by a disproportionality analysis for all unexpectedly identified ADRs not already reported in the summary of product characteristics (SmPCs) available by the European Medicines Agency (EMA) for each considered drug.

## Materials and Methods

### Design of the Study

This was a retrospective observational study in which data were collected through the Italian SRS database called *Rete Nazionale di Farmacovigilanza* (RNF), managed by the Italian Medicines Agency (AIFA). The RNF was established in 2001 with the aim of collecting all suspected ADR reports from drugs and vaccines sent by all Italian regions. As of July 2017, AIFA has organized a publicly available, online system RAM, which allows access to data relative to ADR reports, uploaded into the RNF since 2002, in aggregated form at a national level. The RAM system is an official website that provides public access to the Italian spontaneous reports of suspected ADRs. Only subsets of data from national spontaneous reports are publicly available, taking into account the need to comply with the European Union and Italian Data Protection Policies. Conversely, Regional Pharmacovigilance Centers have full access to the all spontaneous reporting data of their region in the RNF database, including Sicily. Drugs registered in RNF follow the Anatomical Therapeutical Chemical (ATC) classification, while suspected ADRs are grouped according to the Medical Dictionary for Regulatory Activities (MedDRA^®^).

All data of reports recorded from January 2002 through December 2020, with at least one of the following approved molecules for the treatment of MS and reported as suspected, were included in the analysis: immunostimulants (IFN β-1a, IFN β-1b, PEG-IFN β-1a, and GA) and immunosuppressants (NTZ, FNG, TRF, DMF, Alem, and OCZ). Moreover, only cases related to the branded name “Mavenclad^®^” for Cladr were included to avoid therapeutic bias with other available medications having Cladr as an active substance and approved for other indications. Daclizumab was not considered because it was withdrawn from the market in March 2018 and mitoxantrone was withdrawn due to its absence in regional data reports. In order to obtain an overview of ADRs at national level, data related to these drugs were retrieved from the RAM system. Subsequently, a collection of detailed ADR report information, available in the RNF, was carried out for Sicily. Before proceeding with the analysis, literature data and duplicates were excluded.

### Statistical Analysis

Descriptive analyses of national, publicly-available aggregated data and full-access, regional data were performed to assess demographic characteristics and drug-related variables. Analyses were carried out by gender, age, seriousness, and outcome. An ADR was classified as serious when it was life-threatening or fatal, required hospitalization or prolongation of existing hospitalization, caused a persistent or significant disability/incapacity, a congenital anomaly/congenital defect, or was categorized as another serious medical condition based on clinical judgment or Eudravigilance Important Medical Event (IME) list. The ADRs were analyzed at the MedDRA^®^ classification level of System Organ Class (SOC) and Preferred Term (PT) by ordering the individual ADRs in the SOC equivalent and grouping the PT synonyms of the same clinical condition under a single term (see Supplementary Table S1).

A case-by-case analysis of the reports recorded in Sicily was made by taking into account any concomitant drug, comorbidity and information related to the onset time (time to onset, TTO) and the resolution time (time-to- resolution, TTR) of the ADR. The TTO and TTR are expressed in days and were calculated considering the time elapsed between the beginning of the use of the suspected drug and the date of onset of ADR and the time elapsed between the onset of ADR and the resolution of the same ADR, respectively. Furthermore, the causal association between ADR and the suspected drug was evaluated using the Naranjo algorithm and, according to the algorithm score, each ADR was classified as: very likely (score ≥9), probable (scores 5–8), possible (scores 1–4) or doubtful (score ≤0) (Naranjo et al., 1981).

Absolute and relative frequencies with 95% confidence intervals (CI) were evaluated for categorical variables, while medians with interquartile intervals (IQR) were evaluated for continuous variables. Subsequently, a disproportionality analysis was performed for all unexpected ADRs of Sicilian data by checking the SmPCs available at the time of the study on the EMA website. The reporting odds ratio (ROR) and the CIs at 95% were calculated as a measure of disproportionality when the number of these cases was equal to or greater than three. Statistical analyses were conducted using the Statistical Package for the Social Science (SPSS) version 23.0 software for Windows (IBM Corp. SPSS Statistics).

## Results

### Analysis of National Aggregated Data

**FIGURE 1:**
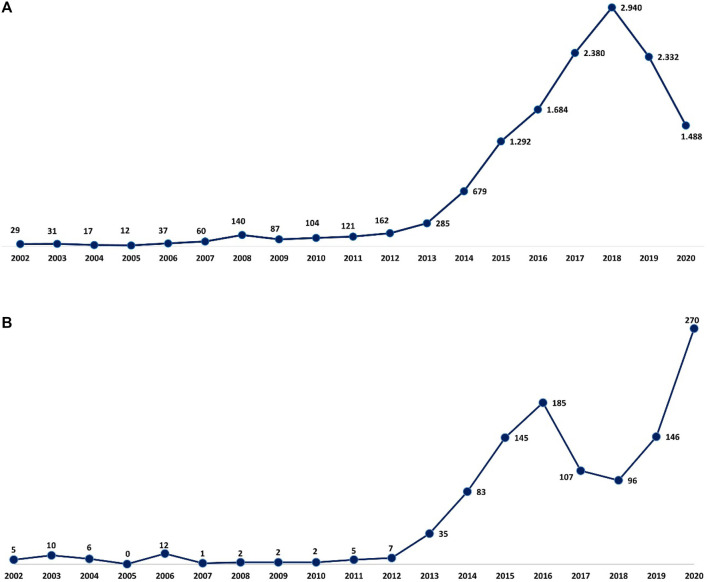
Italian **(A)** and Sicilian **(B)** trend of ADR reports related to drugs approved for MS over the years. ADR, adverse drug reaction; MS, multiple sclerosis.

A total of 13,880 reports of suspected ADRs related to MS drugs have been collected from 2002 to 2020 into the RAM system with a gradual increase over the years, a peak in 2018 (*n* = 2,940, 21.2%), and a decrease in the last 2 years (Figure 1). From 2002 to 2006, approximately 90% of the reports had, as suspected drug, GA and IFN β-1a, while from 2008 to 2012 most reports were attributed to NTZ followed by immunostimulants. From 2013 to 2020, in addition to the high number related to IFN β-1a and GA, a gradual increase of reports was noticed with FNG, Alem, DMF, and TRF. More than half of the 13,880 cases were associated with not serious ADRs (not serious, 67.9% vs. serious, 26.1%). The highest number of serious ADRs was reported for FNG (*n* = 1,112; 41.9%), OCZ (*n* = 138; 40.6%), and NTZ (*n* = 496; 39.2%). Focusing on gender, a larger number of cases was registered in women (females, 71.7% vs. males, 26.3%), and 76.5% of ADRs was reported in the age group of 18–65 years (Table 1).

**TABLE 1:** Descriptions of Italian ADR reports related to drugs for MS collected into the RAM system from January 2002 to December 2020.

Suspected drug	Total	Seriousness			Gender			Age group			
		Serious *n* (%)	Not serious *n* (%)	NA *n* (%)	Female *n* (%)	Male *n* (%)	NA *n* (%)	<18 years *n* (%)	18–65 years *n* (%)	≥66 years *n* (%)	NA *n* (%)
Immunostimulants											
IFN β-1a	3,097	349 (11.3)	2,378 (76.8)	370 (11.9)	2,271 (73.3)	804 (26.0)	22 (0.7)	41 (1.3)	1,666 (53.8)	19 (0.6)	1,313 (42.4)
IFN β-1b	541	80 (14.8)	271 (50.1)	190 (35.1)	391 (72.3)	138 (25.5)	12 (2.2)	2 (0.2)	509 (94.1)	16 (3.0)	15 (2.8)
PEG-IFN β-1a	558	66 (11.8)	461 (82.6)	31 (5.6)	373 (66.8)	102 (18.3)	83 (14.9)		317 (56.8)	3 (0.5)	238 (42.7)
GA	2,047	446 (21.8)	1,562 (76.3)	39 (1.9)	1,588 (77.6)	428 (20.9)	31 (1.5)	14 (0.7)	1,617 (79.0)	41 (2.0)	375 (18.3)
Immunosuppressants											
Alem	1,226	359 (29.3)	851 (69.4)	16 (1.3)	853 (69.6)	357 (29.1)	16 (1.3)	2 (0.2)	1,076 (87.8)	7 (0.6)	141 (11.5)
Cladr	72	13 (18.1)	59 (81.9)		57 (79.2)	15 (20.8)			59 (81.9)		13 (18.1)
DMF	1,326	326 (24.6)	979 (73.8)	21 (1.6)	962 (72.5)	339 (25.6)	25 (1.9)	5 (0.4)	1,067 (80.5)	18 (1.4)	236 (17.8)
FNG	2,652	1,112 (41.9)	1,418 (53.5)	122 (4.6)	1,805 (68.1)	815 (30.7)	32 (1.2)	13 (0.5)	2,391 (90.2)	19 (0.7)	229 (8.6)
NTZ	1,265	496 (39.2)	741 (58.6)	28 (2.2)	923 (73.0)	317 (25.1)	25 (2.0)	13 (1.0)	1,054 (83.3)	48 (3.8)	150 (11.9)
OCZ	340	138 (40.6)	200 (58.8)	2 (0.6)	188 (55.3)	143 (42.1)	9 (2.6)		286 (84.1)	8 (2.4)	46 (13.5)
TRF	756	236 (31.2)	506 (66.9)	14 (1.9)	538 (71.2)	195 (25.8)	23 (3.0)		575 (76.1)	24 (3.2)	157 (20.8)
Total	13,880	3,621 (26.1)	9,426 (67.9)	833 (6.0)	9,949 (71.7)	3,653 (26.3)	278 (2.0)	90 (0.6)	10,617 (76.5)	203 (1.5)	2,913 (21.0)

ADR, adverse drug reaction; Alem, alemtuzumab; Cladr, cladribine; DMF, dimethyl fumarate; FNG, fingolimod; GA, glatiramer acetate; IFN β-1a, interferon β-1a; IFN β-1b, interferon β-1b; MS, multiple sclerosis; NA, not available; NTZ, natalizumab; OCZ, ocrelizumab; PEG-IFN β-1a, peginterferon β-1a; RAM, Reports of Adverse Reactions of Medicines; TRF, teriflunomide.

The most reported ADRs were related to general and administration site conditions (*n* = 6,565; 47.3%), followed by nervous (*n* = 3,090; 22.3%), skin (*n* = 2,763; 19.9%), blood disorders (*n* = 2,180; 15.7%), investigations (*n* = 2,023; 14.6%), infections (*n* = 1,980; 14.3%), and gastrointestinal disorders (*n* = 1,914; 13.8%). General and administration site conditions were predominantly related to immunostimulants (GA: *n* = 1,918; 93.7%, IFN β-1b: *n* = 449; 83%, PEG-IFN β-1a: *n* = 453; 81.2%, and IFN β-1a: *n* = 2,302; 74.3%); PEG-IFN β-1a and IFN β-1b were mostly reported for pyrexia (*n* = 77; 13.8% and *n* = 65; 12%, respectively), while GA was reported for administration site pain (*n* = 313; 15.3%). Moreover, skin disorders, including rash (*n* = 333; 27.2%), were mostly described for Alem. Almost half of Cladr-related ADRs belonged to blood disorders (*n* = 30; 41.7%), especially lymphopenia (*n* = 21; 29.2%), while FNG-related reports mainly showed alteration of liver enzymes (*n* = 1,059; 39.9%) and bradycardia (*n* = 475; 17.9%). A higher number of ADRs related to gastrointestinal disorders, particularly abdominal pain (*n* = 198; 14.9%), nausea (*n* = 77; 5.8%), and diarrhea (*n* = 98; 7.4%) were reported for DMF and TRF. Furthermore, a greater number of nervous disorders were associated with NTZ (*n* = 430; 34%), Cladr (*n* = 24; 33.3%), and Alem (*n* = 404; 33%). Concerning infections, OCZ and Cladr had a higher number of related ADRs (*n* = 109; 32.1% and *n* = 21; 29.2%, respectively). The onset of respiratory disorders, especially dyspnea, was mainly associated with OCZ and GA (*n* = 73; 21.5% and *n* = 321; 15.7%, respectively) while the onset of neoplasms and metabolic disorders was mostly reported for FNG (*n* = 306; 11.5%; *n* = 233; 8.8%, respectively) (Table 2).

**TABLE 2:** Descriptions of Sicilian ADR reports related to drugs for MS collected into the RNF from January 2002 to December 2020.

Suspected drug	Total	Seriousness			Gender			Age group			
		Serious *n* (%)	Not serious *n* (%)	NA *n* (%)	Female *n* (%)	Male *n* (%)	NA *n* (%)	<18 years *n* (%)	18–65 years *n* (%)	≥66 years *n* (%)	NA *n* (%)
Immunostimulants											
IFN β-1a	197	30 (15.2)	136 (69.0)	31 (15.7)	130 (66.0)	65 (33.0)	2 (1.0)	-	168 (85.3)	1 (0.5)	28 (14.2)
IFN β-1b	87	22 (25.3)	48 (55.2)	17 (19.5)	68 (78.2)	18 (20.7)	1 (1.1)	-	81 (93.1)	3 (3.4)	3 (3.4)
PEG-IFN β-1a	38	7 (18.4)	27 (71.1)	4 (10.5)	28 (73.7)	10 (26.3)	-	-	33 (86.8)	-	5 (13.2)
GA	103	25 (24.3)	70 (68.0)	8 (7.8)	73 (70.9)	27 (26.2)	3 (2.9)	1 (1.0)	96 (93.2)	2 (1.9)	4 (3.9)
Immunosuppressants											
Alem	58	15 (25.9)	41 (70.7)	2 (3.4)	38 (65.5)	20 (34.5)	-	-	55 (94.8)	-	3 (5.2)
Cladr	31	5 (16.1)	26 (83.9)	-	29 (93.5)	2 (6.5)	-	-	31 (100.0)	-	-
DMF	124	32 (25.8)	89 (71.8)	3 (2.4)	95 (76.6)	28 (22.6)	1 (0.8)	-	114 (91.9)	-	10 (8.1)
FNG	172	59 (34.3)	108 (62.8)	5 (2.9)	131 (76.2)	40 (23.3)	1 (0.6)	-	166 (96.5)	-	6 (3.5)
NTZ	212	69 (32.5)	138 (65.1)	5 (2.4)	154 (72.6)	55 (25.9)	3 (1.4)	2 (0.9)	192 (90.6)	8 (3.8)	10 (4.7)
OCZ	13	7 (53.8)	6 (46.2)	-	9 (69.2)	4 (30.8)	-	-	11 (84.6)	1 (7.7)	1 (7.7)
TRF	81	23 (28.4)	57 (70.4)	1 (1.2)	52 (64.2)	28 (34.6)	1 (1.2)	-	73 (90.1)	2 (2.5)	6 (7.4)
FNG + DMF or + NTZ	3	3 (100)			2 (66.6)	1 (33.3)			2 (66.6)	1 (33.3)	
Total	1,119	297 (26.5)	746 (66.7)	76 (6.8)	809 (72.3)	298 (26.6)	12 (1.1)	3 (0.3)	1,022 (91.3)	18 (1.6)	76 (6.8)

ADR, adverse drug reaction; Alem, alemtuzumab; Cladr, cladribine; DMF, dimethyl fumarate; FNG, fingolimod; GA, glatiramer acetate; IFN β-1a, interferon β-1a; IFN β-1b, interferon β-1b; MS, multiple sclerosis; NA, not available; NTZ, natalizumab; OCZ, ocrelizumab; PEG-IFN β-1a, peginterferon β-1a; RNF, Rete Nazionale di Farmacovigilanza; TRF, teriflunomide.

### Analysis of Regional Data

Concerning Sicilian data, 1,119 reports were reported of which 3 reports had 2 MS drugs described as suspected and, specifically, 2 cases of fingolimod + dimethyl fumarate and 1 case of fingolimod + natalizumab used in a different period. Different reporting over the years was observed, contrary to Italian reports, and a gradual increase was noticed until 2016, with a decrease in 2017 and 2018 and then another increase in the last 2 years with a peak in 2020 (*n* = 270; 24.2%) (Figure 1). As reported in national data, a higher prevalence of not serious ADRs was noticed for all DMTs except for OCZ (serious, 53.8% vs. not serious, 46.2%). Moreover, a higher number of reports was associated with females, and the 18–65 age group was found to have the most DMT cases (Table 3).

**TABLE 3:** Descriptions of main ADRs associated with drugs for MS treatment and reported into the RAM system.

Adverse drug reaction, *n* (%)	Immunostimulants				Immunosuppressants							Total (*n* = 13,880)
	IFN β-1a (*n* = 3,097)	IFN β-1b (*n* = 541)	PEG-IFN β-1a (*n* = 558)	GA (*n* = 2,047)	Alem (*n* = 1,226)	Cladr (*n* = 72)	DMF (*n* = 1,326)	FNG (*n* = 2,652)	NTZ (*n* = 1,265)	OCZ (*n* = 340)	TRF (*n* = 756)	
General disorders and administration site conditions	2,302 (74.3%)	449 (83%)	453 (81.2%)	1,918 (93.7%)	457 (37.3%)	17 (23.6%)	172 (13%)	365 (13.8%)	186 (14.7%)	115 (33.8%)	131 (17.3%)	6,565
Pyrexia	256 (8.3%)	65 (12%)	77 (13.8%)	109 (5.3%)	263 (21.5%)	6 (8.3%)	34 (2.6%)	55 (2.1%)	40 (3.2%)	31 (9.1%)	25 (3.3%)	961
Asthenia	194 (6.3%)	50 (9.2%)	35 (6.3%)	104 (5.1%)	38 (3.1%)	3 (4.2%)	46 (3.5%)	113 (4.3%)	44 (3.5%)	18 (5.3%)	15 (2%)	660
Administration site pain	226 (7.3%)	45 (8.3%)	17 (3%)	313 (15.3%)	1 (0.1%)				2 (0.2%)			604
Nervous system disorders	622 (20.1%)	122 (22.6%)	83 (14.9%)	486 (23.7%)	404 (33%)	24 (33.3%)	196 (14.8%)	507 (19.1%)	430 (34%)	46 (13.5%)	170 (22.5%)	3,090
Headache	228 (7.4%)	31 (5.7%)	42 (7.5%)	90 (4.4%)	234 (19.1%)	10 (13.9%)	32 (2.4%)	110 (4.1%)	62 (4.9%)	16 (4.7%)	14 (1.9%)	869
MS relapse	131 (4.2%)	32 (5.9%)	10 (1.8%)	102 (5%)	72 (5.9%)	6 (8.3%)	48 (3.6%)	66 (2.5%)	124 (9.8%)	2 (0.6%)	63 (8.3%)	656
Dizziness	26 (0.8%)	1 (0.2%)	6 (1.1%)	27 (1.3%)	10 (0.8%)	2 (2.8%)	11 (0.8%)	21 (0.8%)	29 (2.3%)	5 (1.5%)	1 (0.1%)	139
Skin and subcutaneous tissue disorders	338 (10.9%)	88 (16.3%)	89 (15.9%)	706 (34.5%)	548 (44.7%)	25 (34.7%)	302 (22.8%)	150 (5.7%)	219 (17.3%)	75 (22.1%)	223 (29.5%)	2,763
Rash	82 (2.6%)	38 (7%)	32 (5.7%)	245 (12%)	333 (27.2%)	1 (1.4%)	138 (10.4%)	39 (1.5%)	64 (5.1%)	50 (14.7%)	29 (3.8%)	1,051
Pruritus	30 (1%)	7 (1.3%)	16 (2.9%)	99 (4.8%)	74 (6%)	3 (4.2%)	73 (5.5%)	14 (0.5%)	47 (3.7%)	10 (2.9%)	22 (2.9%)	395
Urticaria	24 (0.8%)	16 (3%)	11 (2%)	148 (7.2%)	47 (3.8%)	2 (2.8%)	35 (2.6%)	4 (0.2%)	55 (4.3%)	4 (1.2%)	8 (1.1%)	354
Blood and lymphatic system disorders	180 (5.8%)	16 (3%)	100 (17.9%)	46 (2.2%)	197 (16.1%)	30 (41.7%)	274 (20.7%)	1,074 (40.5%)	152 (12%)	31 (9.1%)	80 (10.6%)	2,180
Lymphopenia	20 (0.6%)	3 (0.6%)	12 (2.2%)	4 (0.2%)	52 (4.2%)	21 (29.2%)	190 (14.3%)	569 (21.5%)	8 (0.6%)	7 (2.1%)	11 (1.5%)	897
Leukopenia	45 (1.5%)	2 (0.4%)	30 (5.4%)	3 (0.1%)	16 (1.3%)	1 (1.4%)	33 (2.5%)	334 (12.6%)	5 (0.4%)	5 (1.5%)	18 (2.4%)	492
Thrombocytopenia	36 (1.2%)	6 (1.1%)	15 (2.7%)	6 (0.3%)	34 (2.8%)	3 (4.2%)	6 (0.5%)	17 (0.6%)	25 (2%)		15 (2%)	163
Investigations	292 (9.4%)	26 (4.8%)	28 (5%)	70 (3.4%)	191 (15.6%)	10 (13.9%)	100 (7.5%)	1,059 (39.9%)	112 (8.9%)	16 (4.7%)	119 (15.7%)	2,023
Gamma-GT increased	16 (0.5%)	1 (0.2%)	5 (0.9%)	5 (0.2%)	5 (0.4%)		11 (0.8%)	266 (10%)	7 (0.6%)	1 (0.3%)	18 (2.4%)	335
ALT increased	40 (1.3%)	2 (0.4%)	2 (0.4%)	1 (<0.1%)	6 (0.5%)	2 (2.8%)	11 (0.8%)	187 (7.1%)	5 (0.4%)		9 (1.2%)	265
ASP increased	24 (0.8%)	2 (0.4%)	2 (0.4%)	1 (<0.1%)	4 (0.3%)	1 (1.4%)	9 (0.7%)	101 (3.8%)	5 (0.4%)		3 (0.4%)	152
Infections and infestations	181 (5.8%)	45 (8.3%)	56 (10%)	77 (3.8%)	289 (23.6%)	21 (29.2%)	213 (16.1%)	578 (21.8%)	288 (22.8%)	109 (32.1%)	123 (16.3%)	1,980
Herpes zoster	5 (0.2%)		2 (0.4%)	4 (0.2%)	25 (2%)	1 (1.4%)	41 (3.1%)	78 (2.9%)	32 (2.5%)	11 (3.2%)	9 (1.2%)	208
Influenza	68 (2.2%)	14 (2.6%)	40 (7.2%)	13 (0.6%)	17 (1.4%)		3 (0.2%)	39 (1.5%)	3 (0.2%)	1 (0.3%)	3 (0.4%)	201
Viral infection	11 (0.4%)	1 (0.2%)	2 (0.4%)	11 (0.5%)	16 (1.3%)		30 (2.3%)	34 (1.3%)	22 (1.7%)	17 (5%)	16 (2.1%)	160
Progressive multifocal leukoencephalopathy				1 (<0.1%)			4 (5.6%)	7 (0.3%)	96 (7.6%)	2 (0.6%)	3 (0.4%)	113
Gastrointestinal disorders	162 (5.2%)	25 (4.6%)	32 (5.7%)	332 (16.2%)	180 (14.7%)	11 (15.3%)	571 (43.1%)	260 (9.8%)	88 (7%)	58 (17.1%)	195 (25.8%)	1,914
Abdominal pain	16 (0.5%)	5 (0.9%)	3 (0.5%)	65 (3.2%)	33 (2.7%)	2 (2.8%)	198 (14.9%)	38 (1.4%)	9 (0.7%)	7 (2.1%)	28 (3.7%)	404
Nausea	40 (1.3%)	5 (0.9%)	11 (2%)	81 (4%)	50 (4.1%)	2 (2.8%)	77 (5.8%)	44 (1.7%)	21 (1.7%)	11 (3.2%)	20 (2.6%)	362
Diarrhea	12 (0.4%)	4 (0.7%)	2 (0.4%)	30 (1.5%)	19 (1.5%)	3 (4.2%)	98 (7.4%)	37 (1.4%)	12 (0.9%)	5 (1.5%)	70 (9.3%)	292
Cardiac disorders	38 (1.2%)	9 (1.7%)	10 (1.8%)	197 (9.6%)	178 (14.5%)	2 (2.8%)	19 (1.4%)	671 (25.3%)	24 (1.9%)	14 (4.1%)	20 (2.6%)	1,182
Bradycardia				3 (0.1%)	99 (8.1%)			475 (17.9%)	2 (0.2%)			579
Tachycardia	5 (0.2%)	3 (0.6%)	4 (0.7%)	121 (5.9%)	58 (4.7%)	2 (2.8%)	8 (0.6%)	26 (1%)	6 (0.5%)	7 (2.1%)	8 (1.1%)	248
Palpitations	5 (0.2%)	1 (0.2%)		47 (2.3%)	6 (0.5%)		2 (0.2%)	13 (0.5%)			1 (0.1%)	75
Musculoskeletal and connective tissue disorders	334 (10.8%)	84 (15.5%)	86 (15.4%)	164 (8%)	101 (8.2%)	6 (8.3%)	86 (6.5%)	145 (5.5%)	56 (4.4%)	9 (2.6%)	48 (6.3%)	1,119
Myalgia	109 (3.5%)	20 (3.7%)	25 (4.5%)	28 (1.4%)	20 (1.6%)		13 (1%)	11 (0.4%)	4 (0.3%)	2 (0.6%)	6 (0.8%)	238
Arthralgia	61 (2%)	10 (1.8%)	20 (3.6%)	20 (1%)	21 (1.7%)	2 (2.8%)	29 (2.2%)	25 (0.9%)	7 (0.6%)	3 (0.9%)	6 (0.8%)	204
Pain in extremity	44 (1.4%)	14 (2.6%)	4 (0.7%)	29 (1.4%)	15 (1.2%)	1 (1.4%)	8 (0.6%)	14 (0.5%)	3 (0.2%)	1 (0.3%)	11 (1.5%)	144
Respiratory, thoracic and mediastinal disorders	56 (1.8%)	6 (1.1%)	5 (0.9%)	321 (15.7%)	130 (10.6%)	3 (4.2%)	69 (5.2%)	121 (4.6%)	69 (5.5%)	73 (21.5%)	40 (5.3%)	893
Dyspnoea	17 (0.5%)	2 (0.4%)	4 (0.7%)	194 (9.5%)	22 (1.8%)		16 (1.2%)	25 (0.9%)	19 (1.5%)	11 (3.2%)	8 (1.1%)	318
Cough	9 (0.3%)	1 (0.2%)		15 (0.7%)	28 (2.3%)	1 (1.4%)	23 (1.7%)	32 (1.2%)	11 (0.9%)	8 (2.4%)	10 (1.3%)	138
Choking	1 (<0.1%)			37 (1.8%)	1 (0.1%)		2 (0.2%)		2 (0.2%)			43
Injury, poisoning and procedural complications	243 (7.8%)	33 (6.1%)	51 (9.1%)	176 (8.6%)	38 (3.1%)	8 (11.1%)	33 (2.5%)	154 (5.8%)	72 (5.7%)	13 (3.8%)	14 (1.9%)	835
Administration related reaction	51 (1.6%)	22 (4.1%)	25 (4.5%)	102 (5%)	2 (0.2%)				4 (0.3%)	2 (0.6%)		208
Maternal exposure during pregnancy	8 (0.3%)		1 (0.2%)	5 (0.2%)			8 (0.6%)	39 (1.5%)	22 (1.7%)			83
Skin wound	3 (0.1%)	1 (0.2%)	1 (0.2%)	6 (0.3%)	6 (0.5%)		2 (0.2%)	24 (0.9%)	2 (0.2%)	3 (0.9%)		48
Vascular disorders	70 (2.3%)	19 (3.5%)	6 (1.1%)	159 (7.8%)	60 (4.9%)	1 (1.4%)	174 (13.1%)	124 (4.7%)	40 (3.2%)	19 (5.6%)	71 (9.4%)	743
Flushing	10 (0.3%)	4 (0.7%)	1 (0.2%)	76 (3.7%)	12 (1%)	1 (1.4%)	140 (10.6%)	5 (0.2%)	4 (0.3%)	2 (0.6%)	1 (0.1%)	256
Hypertension	18 (0.6%)	5 (0.9%)	3 (0.5%)	7 (0.3%)	17 (1.4%)		2 (0.2%)	66 (2.5%)	6 (0.5%)	3 (0.9%)	65 (8.6%)	192
Hot flushes	14 (0.5%)	3 (0.6%)	2 (0.4%)	97 (4.7%)	9 (0.7%)		34 (2.6%)	7 (0.3%)	7 (0.6%)	2 (0.6%)		175
Neoplasm bening, malignant and unspecified	107 (3.5%)	10 (1.8%)	2 (0.4%)	35 (1.7%)	18 (1.5%)	5 (6.9%)	68 (5.1%)	306 (11.5%)	100 (7.9%)	13 (3.8%)	51 (6.7%)	715
Breast cancer	7 (0.2%)	3 (0.6%)		5 (0.2%)	4 (0.3%)		18 (1.4%)	30 (1.1%)	20 (1.6%)	4 (1.2%)	3 (0.4%)	94
Malignant melanoma	8 (0.3%)	1 (0.2%)		1 (<0.1%)		2 (2.8%)	8 (0.6%)	47 (1.8%)	12 (0.9%)	1 (0.3%)	1 (0.1%)	81
Basal cell carcinoma				6 (0.3%)				48 (1.8%)	2 (0.2%)	1 (0.3%)	1 (0.1%)	58
Psychiatric disorders	288 (9.3%)	30 (5.5%)	31 (5.6%)	84 (4.1%)	54 (4.4%)		33 (2.5%)	115 (4.3%)	37 (2.9%)	4 (1.2%)	17 (2.2%)	693
Insomnia	40 (1.3%)	6 (1.1%)	6 (1.1%)	9 (0.4%)	23 (1.9%)		7 (0.5%)	22 (0.8%)	2 (0.2%)		1 (0.1%)	116
Depression	28 (0.9%)	7 (1.3%)	5 (0.9%)	13 (0.6%)	7 (0.6%)		3 (0.2%)	30 (1.1%)	5 (0.4%)		1 (0.1%)	99
Anxiety	32 (1%)	3 (0.6%)	1 (0.2%)	12 (0.6%)	11 (0.9%)		1 (0.1%)	23 (0.9%)	1 (0.1%)		7 (0.9%)	91
Hepatobiliary disorders	68 (2.2%)	16 (3%)	10 (1.8%)	24 (1.2%)	25 (2%)	2 (2.8%)	49 (3.7%)	170 (6.4%)	42 (3.3%)	5 (1.5%)	41 (5.4%)	452
Hypertransaminasaemia	77 (2.5%)	10 (1.8%)	10 (1.8%)	17 (0.8%)	14 (1.1%)	2 (2.8%)	41 (3.1%)	121 (4.6%)	24 (1.9%)	2 (0.6%)	43 (5.7%)	361
Hyperbilirubinaemia	11 (0.4%)		1 (0.2%)		3 (0.2%)	2 (2.8%)	4 (0.3%)	57 (2.1%)	6 (0.5%)	1 (0.3%)	3 (0.4%)	88
Liver injury	17 (0.5%)	3 (0.6%)	2 (0.4%)	4 (0.2%)	2 (0.2%)		7 (0.5%)	17 (0.6%)	15 (1.2%)		6 (0.8%)	73
Metabolism and nutrition disorders	38 (1.2%)	7 (1.3%)	7 (1.3%)	19 (0.9%)	30 (2.4%)		24 (1.8%)	233 (8.8%)	17 (1.3%)	4 (1.2%)	22 (2.9%)	401
Dyslipidaemia	9 (0.3%)		3 (0.5%)	2 (0.1%)	4 (0.3%)		2 (0.2%)	170 (6.4%)	6 (0.5%)	1 (0.3%)	6 (0.8%)	203
Decreased appetite	18 (0.6%)	2 (0.4%)	1 (0.2%)	4 (0.2%)			8 (0.6%)	9 (0.3%)	1 (0.1%)		10 (1.3%)	53
Vitamin D deficiency	2 (0.1%)	2 (0.4%)		3 (0.1%)	17 (1.4%)		1 (0.1%)	24 (0.9%)			3 (0.4%)	52
Eye disorders	96 (3.1%)	10 (1.8%)	2 (0.4%)	32 (1.6%)	26 (2.1%)	4 (5.6%)	22 (1.7%)	96 (3.6%)	35 (2.8%)	9 (2.6%)	6 (0.8%)	338
Vision blurred	9 (0.3%)	3 (0.6%)		4 (0.2%)	1 (0.1%)	2 (2.8%)	6 (0.5%)	6 (0.2%)	11 (0.9%)	2 (0.6%)		44
Visual impairment	13 (0.4%)	4 (0.7%)		2 (0.1%)	3 (0.2%)		1 (0.1%)	11 (0.4%)	7 (0.6%)		2 (0.3%)	43
Visual acuity reduced	28 (0.9%)			1 (<0.1%)	1 (0.1%)			5 (0.2%)				35
Pregnancy, puerperium and perinatal conditions	53 (1.7%)	1 (0.2%)	3 (0.5%)	15 (0.7%)	27 (2.2%)	4 (5.6%)	15 (1.1%)	67 (2.5%)	46 (3.6%)	3 (0.9%)	4 (0.5%)	238
Abortion	47 (1.5%)		1 (0.2%)	6 (0.3%)	8 (0.7%)	2 (2.8%)	13 (1%)	17 (0.6%)	25 (2%)	1 (0.3%)	4 (0.5%)	124
Normal newborn	2 (0.1%)			1 (<0.1%)	1 (0.1%)			20 (0.8%)				24
Pregnancy		1 (0.2%)	1 (0.2%)		14 (1.1%)		1 (0.1%)	4 (0.2%)	1 (0.1%)	1 (0.3%)		23
Reproductive system and breast disorders	42 (1.4%)	1 (0.2%)	4 (0.7%)	20 (1%)	24 (2%)		22 (1.7%)	77 (2.9%)	19 (1.5%)	8 (2.4%)	18 (2.4%)	235
Amenorrhoea	15 (0.5%)			4 (0.2%)	1 (0.1%)		4 (0.3%)	5 (0.2%)			2 (0.3%)	31
Cervical dysplasia				2 (0.1%)	4 (0.3%)		3 (0.2%)	14 (0.5%)	6 (0.5%)			29
Dysmenorrhoea	4 (0.1%)				4 (0.3%)		1 (0.1%)	3 (0.1%)			2 (0.3%)	14
Renal and urinary disorders	59 (1.9%)	7 (1.3%)	5 (0.9%)	22 (1.1%)	28 (2.3%)		13 (1%)	61 (2.3%)	16 (1.3%)	6 (1.8%)	5 (0.7%)	222
Proteinuria	13 (0.4%)				8 (0.7%)			2 (0.1%)	1 (0.1%)		1 (0.1%)	25
Micturition urgency				3 (0.1%)				14 (0.5%)				17
Renal colic	5 (0.2%)			2 (0.1%)			2 (0.2%)	6 (0.2%)	1 (0.1%)			16
Immune system disorders	9 (0.3%)	2 (0.4%)	3 (0.5%)	92 (4.5%)	20 (1.6%)		14 (1.1%)	11 (0.4%)	42 (3.3%)	7 (2.1%)	7 (0.9%)	207
Hypersensitivity	9 (0.3%)	2 (0.4%)	2 (0.4%)	73 (3.6%)	12 (1%)		12 (0.9%)	5 (0.2%)	15 (1.2%)	1 (0.3%)	6 (0.8%)	137
Anaphylactic shock				11 (0.5%)	1 (0.1%)		1 (0.1%)	1 (<0.1%)	4 (0.3%)			18
Anaphylactic reaction				3 (0.1%)	1 (0.1%)				11 (0.9%)	1 (0.3%)		16
Endocrine disorders	20 (0.6%)	6 (1.1%)		1 (<0.1%)	142 (11.6%)	3 (4.2%)	1 (0.1%)	14 (0.5%)	1 (0.1%)	1 (0.3%)	4 (0.5%)	193
Autoimmune thyroiditis	1 (<0.1%)	2 (0.4%)			41 (3.3%)			1 (<0.1%)				45
Hypothyroidism	7 (0.2%)	2 (0.4%)			23 (1.9%)	2 (2.8%)		2 (0.1%)				36
Hyperthyroidism		1 (0.2%)		1 (<0.1%)	25 (2%)			3 (0.1%)		1 (0.3%)	1 (0.1%)	32

ADR, adverse drug reaction; Alem, alemtuzumab; ALT, alanine aminotransferase; ASP, aspartate aminotransferase; Cladr, cladribine; DMF, dimethyl fumarate; FNG, fingolimod; GA, glatiramer acetate; Gamma-GT, gamma-glutamyltransferase; IFN β-1a, interferon β-1a; IFN β-1b, interferon β-1b; MS, multiple sclerosis; NTZ, natalizumab; OCZ, ocrelizumab; PEG-IFN β-1a, peginterferon β-1a; RAM, Reports of Adverse Reactions of Medicines; TRF, teriflunomide.

**FIGURE 2:**
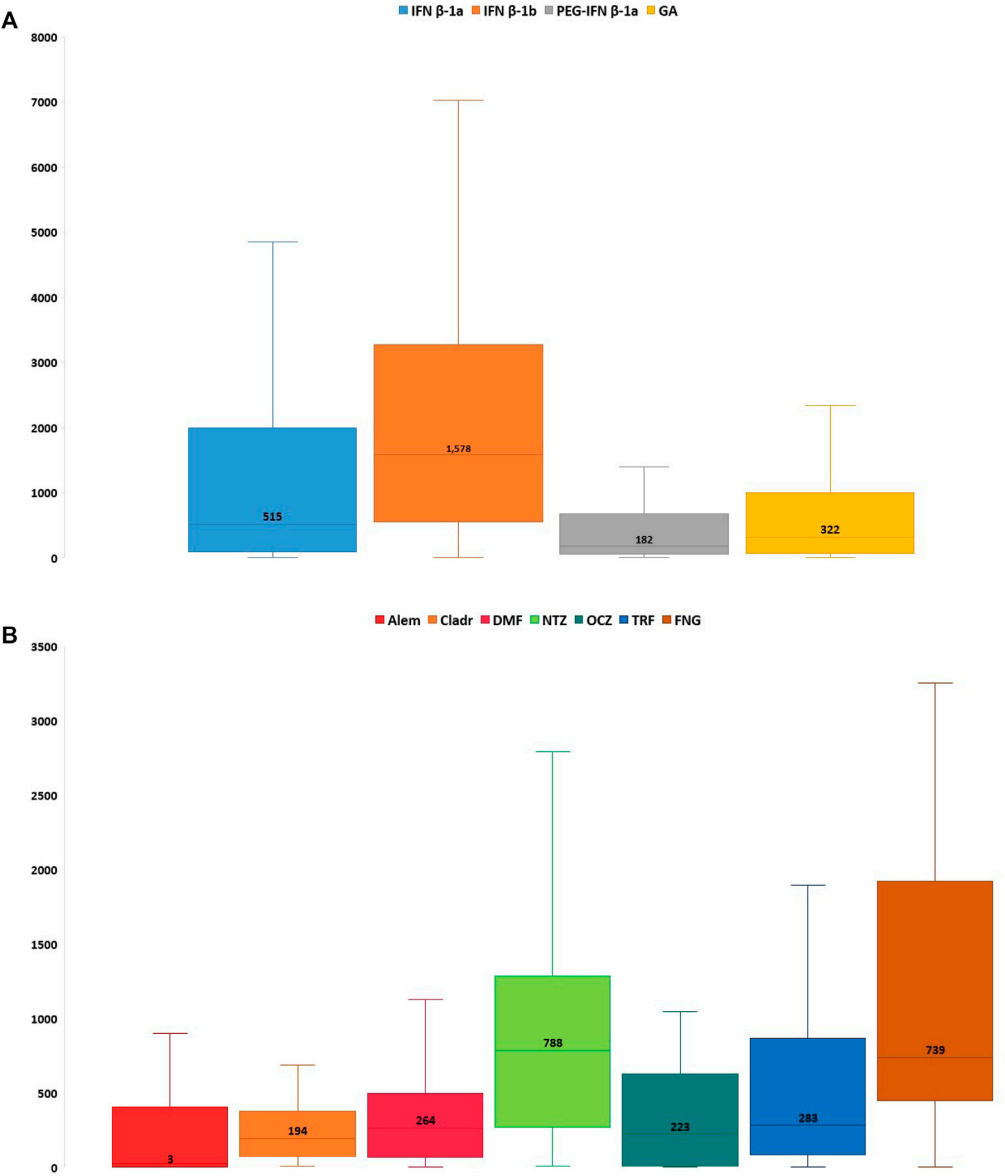
Time to onset of Sicilian ADRs related to immunostimulants **(A)** and immunosuppressants **(B)** approved for MS. ADR, adverse drug reaction; Alem, alemtuzumab; Cladr, cladribine; DMF, dimethyl fumarate; FNG, fingolimod; GA, glatiramer acetate; IFN β-1a, interferon β-1a; IFN β-1b, interferon β-1b; MS, multiple sclerosis; NTZ, natalizumab; OCZ, ocrelizumab; PEG-IFN β-1a, peginterferon β-1a; TRF, teriflunomide.

Considering all reports with only one DMT reported as suspected, the median TTO of ADRs was almost equal for all drugs (from 182 to 322 days) excluding Alem which was related to a median (Q1-Q3) TTO of 27 (3–366) days and IFN β-1b with a median (Q1-Q3) TTO of 1,578 (709–3,094) days (Figure 2). Analyzing Sicilian data, the reported ADRs were substantially equal to national reports, even if other relevant ADRs were the onset of lymphopenia also with FNG and DMF (*n* = 49; 28.5% and *n* = 32; 25.8%, respectively), liver enzymes alteration and the onset of PML with NTZ (*n* = 28; 13.2%; *n* = 12; 5.7%, respectively) (Table 4).

**TABLE 4:** Descriptions of main Sicilian ADRs associated with drugs for MS treatment and reported into the RNF.

Adverse drug reaction, *n* (%)	Immunostimulants				Immunosuppressants								Total (*n* = 1,119)
	IFN β-1a (*n* = 197)	IFN β-1b (*n* = 87)	PEG-IFN β-1a (*n* = 38)	GA (*n* = 103)	Alem (*n* = 58)	Cladr (*n* = 31)	DMF (*n* = 124)	FNG (*n* = 172)	NTZ (*n* = 212)	OCZ (*n* = 13)	TRF (*n* = 81)	FNG + DMF or + NTZ (*n* = 3)	
General disorders and administration site conditions	73 (37.1%)	36 (41.4%)	15 (39.5%)	34 (33%)	12 (20.7%)	1 (3.2%)	12 (9.7%)	13 (7.6%)	18 (8.5%)		5 (6.2%)		219
Pyrexia	14 (7.1%)	11 (12.6%)	5 (13.2%)	4 (3.9%)	8 (13.8%)			2 (1.2%)	2 (0.9%)		2 (2.5%)		48
Influenza like illness	19 (9.6%)	2 (2.3%)	5 (13.2%)										26
Administration site pain	10 (5.1%)	5 (5.7%)	2 (5.3%)	7 (6.8%)									24
Nervous system disorders	53 (26.9%)	19 (21.8%)	5 (13.2%)	31 (30.1%)	10 (17.2%)	3 (9.7%)	13 (10.5%)	14 (8.1%)	25 (11.8%)		14 (17.3%)	1 (33.3%)	188
MS relapse	33 (16.8%)	13 (14.9%)	1 (2.6%)	20 (19.4%)		2 (6.5%)	11 (8.9%)	8 (4.7%)	13 (6.1%)		13 (16%)		114
Headache	15 (7.6%)	2 (2.3%)	2 (5.3%)	3 (2.9%)	7 (12.1%)	1 (3.2%)	2 (1.6%)	1 (0.6%)	4 (1.9%)		1 (1.2%)		38
Paresthesia	1 (0.5%)	2 (2.3%)		2 (1.9%)					2 (0.9%)				7
Blood and lymphatic system disorders	10 (5.1%)	4 (4.6%)	2 (5.3%)	1 (1%)	9 (15.5%)	19 (61.3%)	36 (29%)	57 (33.1%)	29 (13.7%)	1 (7.7%)	5 (6.2%)		173
Lymphopenia	3 (1.5%)		1 (2.6%)		3 (5.2%)	16 (51.6%)	32 (25.8%)	49 (28.5%)	1 (0.5%)				105
Leukopenia	1 (0.5%)	1 (1.1%)	1 (2.6%)	1 (1%)		2 (6.5%)	9 (7.3%)	9 (5.2%)			3 (3.7%)		27
Thrombocytopenia	5 (2.5%)				2 (3.4%)	2 (6.5%)			10 (4.7%)		3 (3.7%)		22
Skin and subcutaneous tissue disorders	17 (8.6%)	9 (10.3%)	5 (13.2%)	24 (23.3%)	18 (31%)	1 (3.2%)	12 (9.7%)	9 (5.2%)	23 (10.8%)	1 (7.7%)	11 (13.6%)		130
Rash	5 (2.5%)	7 (8%)	1 (2.6%)	8 (7.8%)	14 (24.1%)		6 (4.8%)	1 (0.6%)	7 (3.3%)		1 (1.2%)		50
Pruritus	2 (1%)	2 (2.3%)	2 (5.3%)	6 (5.8%)	4 (6.9%)		4 (3.2%)		7 (3.3%)	1 (7.7%)	1 (1.2%)		29
Urticaria	5 (2.5%)		1 (2.6%)	6 (5.8%)	2 (3.4%)		1 (0.8%)		9 (4.2%)				24
Infections and infestations	11 (5.6%)	5 (5.7%)	5 (13.2%)	1 (1%)	4 (6.9%)	4 (12.9%)	7 (5.6%)	25 (14.5%)	45 (21.2%)	4 (30.8%)	6 (7.4%)		117
Urinary tract infection	2 (1%)	1 (1.1%)				1 (3.2%)		3 (1.7%)	11 (5.2%)	2 (15.4%)	1 (1.2%)		21
Herpes zoster			1 (2.6%)		2 (3.4%)		3 (2.4%)	4 (2.3%)	5 (2.4%)	1 (7.7%)	1 (1.2%)		17
Progressive multifocal leukoencephalopathy									12 (5.7%)		1 (1.2%)		13
Gastrointestinal disorders	8 (4.1%)	1 (1.1%)	3 (7.9%)	12 (11.7%)	8 (13.8%)	1 (3.2%)	21 (16.9%)	11 (6.4%)	7 (3.3%)	3 (23.1%)	6 (7.4%)		81
Nausea	4 (2%)	1 (1.1%)	2 (5.3%)	3 (2.9%)	6 (10.3%)	1 (3.2%)	5 (4%)	3 (1.7%)	1 (0.5%)	1 (7.7%)			27
Vomiting			2 (5.3%)	4 (3.9%)	3 (5.2%)	1 (3.2%)	6 (4.8%)	2 (1.2%)	2 (0.9%)	1 (7.7%)			21
Abdominal pain				1 (1%)			11 (8.9%)		2 (0.9%)				14
Investigations	7 (3.6%)	2 (2.3%)	3 (7.9%)	3 (2.9%)	2 (3.4%)		5 (4%)	20 (11.6%)	28 (13.2%)		4 (4.9%)		74
Gamma-GT increased	3 (1.5%)		1 (2.6%)		1 (1.7%)		3 (2.4%)	12 (7%)	5 (2.4%)		2 (2.5%)		27
ALT increased			2 (5.3%)	1 (1%)	1 (1.7%)		3 (2.4%)	1 (0.6%)	3 (1.4%)		1 (1.2%)		12
JC polyomavirus test positive									9 (4.2%)				9
Hepatobiliary disorders	9 (4.6%)	4 (4.6%)	6 (15.8%)	4 (3.9%)	1 (1.7%)		11 (8.9%)	12 (7%)	10 (4.7%)		9 (11.1%)		66
Hypertransaminasaemia	6 (3%)	2 (2.3%)	4 (10.5%)	1 (1%)	1 (1.7%)		5 (4%)	7 (4.1%)	6 (2.8%)		9 (11.1%)		41
Hyperbilirubinaemia								3 (1.7%)	4 (1.9%)		1 (1.2%)		8
Liver injury	1 (0.5%)	1 (1.1%)	1 (2.6%)				2 (1.6%)		1 (0.5%)				6
Metabolism and nutrition disorders	11 (5.6%)	2 (2.3%)	1 (2.6%)	4 (3.9%)	2 (3.4%)		6 (4.8%)	16 (9.3%)	9 (4.2%)	1 (7.7%)	4 (4.9%)		56
Dyslipidaemia	4 (2%)		2 (5.3%)		2 (3.4%)		1 (0.8%)	10 (5.8%)	5 (2.4%)	1 (7.7%)	3 (3.7%)		28
Vitamin D deficiency	2 (1%)	2 (2.3%)		3 (2.9%)			1 (0.8%)	1 (0.6%)			1 (1.2%)		10
Vascular disorders	8 (4.1%)	4 (4.6%)	1 (2.6%)	10 (9.7%)	1 (1.7%)		7 (5.6%)	3 (1.7%)	4 (1.9%)	2 (15.4%)	14 (17.3%)		54
Hypertension	1 (0.5%)	1 (1.1%)						2 (1.2%)		1 (7.7%)	13 (16%)		18
Hot flushes	1 (0.5%)		1 (2.6%)	6 (5.8%)			2 (1.6%)		3 (1.4%)				13
Flushing	1 (0.5%)		1 (2.6%)	4 (3.9%)			5 (4%)		1 (0.5%)				12
Neoplasm benign, malignant and unspecified	1 (0.5%)	5 (5.7%)		4 (3.9%)			10 (8.1%)	14 (8.1%)	8 (3.8%)		4 (4.9%)	2 (66.6%)	48
Breast cancer		1 (1.1%)					4 (3.2%)	2 (1.2%)	2 (0.9%)			1 (33.3%)	10
Naevus								3 (1.7%)			1 (1.2%)		4
Lung cancer				1 (1%)				1 (0.6%)	1 (0.5%)		1 (1.2%)		4
Cardiac disorders	1 (0.5%)	2 (2.3%)	1 (2.6%)	9 (8.7%)	5 (8.6%)			15 (8.7%)	6 (2.8%)		3 (3.7%)		42
Tachycardia				7 (6.8%)	4 (6.9%)				2 (0.9%)		1 (1.2%)		14
Bradycardia								4 (2.3%)					4
Extrasystoles								3 (1.7%)					3
Respiratory, thoracic and mediastinal disorders	2 (1%)			13 (12.6%)	7 (12.1%)		2 (1.6%)	5 (2.9%)	9 (4.2%)	1 (7.7%)			39
Dyspnoea	1 (0.5%)			12 (11.7%)	1 (1.7%)		1 (0.8%)	2 (1.2%)	2 (0.9%)				19
Cough				1 (1%)	4 (6.9%)		1 (0.8%)	1 (0.6%)	2 (0.9%)				9
Pneumonitis	1 (0.5%)				2 (3.4%)			1 (0.6%)	1 (0.5%)				5
Musculoskeletal and connective tissue disorders	16 (8.1%)	7 (8%)	3 (7.9%)	1 (1%)	1 (1.7%)		1 (0.8%)	4 (2.3%)	2 (0.9%)		2 (2.5%)		37
Myalgia	7 (3.6%)	3 (3.4%)					1 (0.8%)						11
Arthralgia	1 (0.5%)		3 (7.9%)				1 (0.8%)	1 (0.6%)			1 (1.2%)		7
Pain in extremity	3 (1.5%)							1 (0.6%)					4
Psychiatric disorders	7 (3.6%)	5 (5.7%)	4 (10.5%)	1 (1%)	1 (1.7%)		2 (1.6%)	1 (0.6%)	5 (2.4%)				26
Depression	3 (1.5%)	3 (3.4%)							1 (0.5%)				7
Insomnia	2 (1%)	1 (1.1%)	1 (2.6%)		1 (1.7%)				1 (0.5%)				6
Anxiety		2 (2.3%)						1 (0.6%)	1 (0.5%)				4
Eye disorders	2 (1%)	2 (2.3%)		4 (3.9%)	1 (1.7%)	1 (3.2%)		6 (3.5%)	3 (1.4%)				19
Eye oedema				1 (1%)				3 (1.7%)					4
Visual blurred		1 (1.1%)							1 (0.5%)				2
Exophthalmos	1 (0.5%)			1 (1%)									2
Endocrine disorders	3 (1.5%)	3 (3.4%)			5 (8.6%)	3 (9.7%)	1 (0.8%)	1 (0.6%)	1 (0.5%)		2 (2.5%)		19
Thyroiditis					2 (3.4%)	1 (3.2%)	1 (0.8%)				2 (2.5%)		6
Hypothyroidism	1 (0.5%)	1 (1.1%)				2 (6.5%)							4
Autoimmune thyroiditis					3 (5.2%)								3
Immune system disorders	1 (0.5%)			8 (7.8%)			1 (0.8%)		6 (2.8%)	1 (7.7%)			17
Hypersensitivity	1 (0.5%)			6 (5.8%)			1 (0.8%)		3 (1.4%)				11
Anaphylactic shock				1 (1%)					1 (0.5%)				2
Anaphylactic reaction				1 (1%)					1 (0.5%)				2
Reproductive system and breast disorders	2 (1%)			2 (1.9%)	1 (1.7%)		4 (3.2%)	3 (1.7%)	1 (0.5%)		2 (2.5%)		15
Endometriosis				1 (1%)			1 (0.8%)						2
Breast mass								1 (0.6%)			1 (1.2%)		2
Pregnancy, puerperium and perinatal conditions							1 (0.8%)		11 (5.2%)				12
Abortion							1 (0.8%)		9 (4.2%)				10

ADR, adverse drug reaction; Alem, alemtuzumab; ALT, alanine aminotransferase; ASP, aspartate aminotransferase; Cladr, cladribine; DMF, dimethyl fumarate; FNG, fingolimod; GA, glatiramer acetate; Gamma-GT, gamma-glutamyltransferase; IFN β-1a, interferon β-1a; IFN β-1b, interferon β-1b; MS, multiple sclerosis; NTZ, natalizumab; OCZ, ocrelizumab; PEG-IFN β-1a, peginterferon β-1a; RNF, Rete Nazionale di Farmacovigilanza; TRF, teriflunomide.

The case-by-case assessment of Sicilian ADRs related to life-threatening events, persistent or significant disabilities and death was reported in Table 5. Among these reports, the majority concerned life threatening events (*n* = 11; 10.8%), followed by death and persistent or significant disabilities (*n* = 6; 0.5% and *n* = 2; 0.2%, respectively) with a median TTO of 240 (98.5–774) days. The analyzed reports concerned almost equally female and male patients (10 cases vs. 9 cases) and the median age of subjects was 46 (39–52.5) years. Most reported outcomes for life–threatening ADRs were fully recovered and not yet recovered and were especially related to the onset of breast cancer and skin disorders. Cases of death showed a completed suicide with IFN β-1b and the use of concomitant duloxetine for major depression, a cardiac arrest with FNG and cannabinoids, a cardiorespiratory arrest with Alem, a septic shock under the use of Alem, and a COVID-19 infection with FNG. Reports with a persistent or significant disability were related to a hepatic enzyme increase and to testicular infarction that led to a recovery with sequelae (Table 5).

**TABLE 5:** Sicilian ADR reports related to drugs for MS with life-threatening events, persistent or significant disabilities and death.

Case	Type of seriousness	Age (years)	Sex	Suspected drug(s) (therapeutic indication)	Concomitant drug(s) (therapeutic indication)	ADR(s)	TTO (days)	Outcome	Causality assessment
1	Life-threatening	39	F	Interferon β-1a (MS)	Paracetamol	Asthenia, jaundice, urticaria	58	Non yet recovered	Possible
2	Life-threatening	27	M	Interferon β-1a (MS)	—	Dyspepsia	196	Unknown	Probable
3	Persistent or significant disability	48	M	Interferon β-1a (MS)	—	Hepatic enzyme increased	991	Unknown	Probable
4	Life-threatening	39	F	Glatiramer acetate (MS)	—	Anaphylactic reaction	299	Fully recovered	Probable
5	Life-threatening	46	F	Interferon β-1a (RRMS)	—	Hepatitis fulminant	853	Unknown	Probable
6	Life-threatening	39	M	Glatiramer acetate	—	Dyspnoea, lip oedema, urticaria	1	Unknown	Probable
7	Death	56	F	Interferon β-1b (MS)	Duloxetine hydrochloride (major depression)	Completed suicide	240	Death	Possible
8	Life-threatening	48	F	Glatiramer acetate (MS)	—	Rash	1	Improved	Possible
9	Death	74	M	Natalizumab (RRMS)	Gabapentin	Death	184	Death	Possible
10	Life-threatening	46	F	Natalizumab (MS)	Venlafaxine, acetylsalicylic acid, amantadine, baclofen	Henoch-Schonlein purpura, pruritus, tachycardia, urticaria	184	Fully recovered	Possible
11	Life-threatening	49	M	Natalizumab (RRMS)	—	Metastases to bone, metastases to abdominal wall, lung cancer	900	Unknown	Possible
12	Persistent or significant disability	34	M	Interferon β-1a (RRMS), paracetamol (flu syndrome), tamsulosine (neurogenic bladder)	Clonazepam	Pyrexia, testicular infarction, testicular swelling	410	Recovered with sequelae	Possible
13	Life-threatening	44	F	Fingolimod (MS)	—	Ovarian cancer	139	Unknown	Possible
14	Death	56	M	Fingolimod (MS) and Δ-9-tetrahydrocannabinol with cannabidiol (muscle spasms)	—	Cardiac arrest	1,948	Death	Possible
15	Death	29	F	Alemtuzumab (MS)	Paracetamol and codeine (back pain)	Altered state of consciousness, back pain, cardiac arrest, respiratory arrest	1.214	Death	Possible
16	Life-threatening	38	F	Fingolimod (MS)	Tolterodine	Breast cancer	654	Not yet recovered	Possible
17	Death	46	F	Alemtuzumab (MS)	—	Lymphopenia, pulmonary oedema, pyelonephritis, septic shock	898	Death	Probable
18	Life-threatening	70	M	Natalizumab (MS)	—	Aortic aneurysm, coronary artery disease, aortic valve disease	2,069	Unknown	Possible
19	Death	64	M	Fingolimod (MS)	—	Acute respiratory distress syndrome, COVID-19	695	Death	Probable

ADR, adverse drug reaction; MS, multiple sclerosis; RRMS, relapsing remitting MS; TTO, time to onset.

The disproportionality analysis resulted in some unexpected ADRs including dyslipidemia which was mainly reported for FNG (*n* = 10; ROR 28.5, CI 14.3–59.6), NTZ (*n* = 5; 10.3, 4.1–25.8), and IFN β-1a (*n* = 4; 8.7, 3.1–24.1), and abortion reported for NTZ (*n* = 9; 208.1, 73.4–590.1). Moreover, some cases of unexpected malignancies including lung cancer and prostate cancer were shown for DMF, FNG, NTZ, and GA (all with *n* = 1); in particular, breast cancer was mostly reported for DMF [*n* = 5, of which one in association with FNG; 78.5 (28.1–219.3)], FNG (*n* = 3, of which one in association with DMF; 30.7 (8.9–106.4), and NTZ (both *n* = 2). Vitamin D deficiency was unknown for GA (*n* = 3; 121.2, 30.9–475.3), but also for IFN β-1a, IFN β-1b (both *n* = 2), FNG, and TRF (both *n* = 1). The analyzed reports also listed unexpected alopecia for NTZ (*n* = 3; 4.9, 1.5–15.7) and GA (*n* = 2). Some thyroid disorders were also unknown for Cladr, TRF, DMF, and included especially hypothyroidism with Cladr (*n* = 3; 89.2, 25.9–307.5) and thyroiditis with TRF (*n* = 2), Cladr and DMF (both *n* = 1).

## Discussion

This study aimed to describe the frequency of ADRs collected in the Italian SRS database, focusing on Sicilian ADR reports associated with the use of DMTs for the treatment of MS. A different reporting trend at the national and regional level was shown in our study: with a gradual increase of reporting observed in Italy from 2014 to 2018, while MS drugs-related ADR reports peaked in Sicily between 2016 and 2020. This data are certainly influenced by several observational studies carried out at national or regional level in different periods during the last few years for evaluating the safety of MS drugs including the study BREMSO—BIIT 0114 for IFN β-1a, the study LEMTRADA PASS OBS13434 for Alem, the study FTY720D for FNG and some active pharmacovigilance projects, resulting in several solicited ADR reports in addition to the spontaneous studies in specific Italian regions. Most national and regional ADR reports concerned women (F/M ratio: 2.6) and adult patients. This probably reflects the prevalence of MS, which mostly occurs in patients aged 20–40 years and affects women three times more often than men (Filippi et al., 2018). The ADRs resolved completely or improved in over 40% of cases. Almost 25% of the reports concerned serious ADRs, but analyzing the reporting of individual drugs, a higher frequency of serious ADRs was reported for immunomodulators, including FNG, OCZ, and NTZ as shown in some studies (Hart and Bainbridge, 2016; Li et al., 2020).

With regard to each single type of ADR, our study confirmed well-known MS drug-related safety issues such as injection site reactions with immunostimulants, suggesting patient non-compliance, and the need to more closely monitor the subcutaneous administration for a greater therapeutic adherence (Stewart and Tran, 2012; Aviv et al., 2018; Maurelli et al., 2018). Moreover, a higher occurrence of reports with known skin reactions was shown for Alem (Vukusic et al., 2019), while a higher frequency of lymphopenia and infections was observed for Cladr (Leist et al., 2020). Lymphopenia is a long-known ADR linked to the mechanism of action of Cladr that is preferentially activated inside the B and T lymphocytes interfering with the synthesis of DNA: Cladr is an analogue of deoxyadenosine and, for its resistance to adenosine deaminase (ADA)-mediated deamination, it consequently accumulates in the lymphocytes and determines the formation of the cytotoxic triphosphorylated Cladr which causes ADA deficiency and leads to the development of severe lymphopenia (Baker et al., 2019). Lymphopenia could be associated with the higher onset of infections in patients treated with Cladr due to the immunodeficiency and for this reason, it would be advisable to check the immunological status of patients (Warny et al., 2018). Concerning FNG, reports showed a higher frequency of alteration of liver enzymes and cardiac arrhythmia as observed in the recent study of phase III FREEDOMS II (Calabresi et al., 2014): cardiac side effects with FNG are probably related to transient agonism to sphingosine-1-phosphate receptors on cardiomyocytes (Forrest et al., 2004). According to other results, OCZ was mainly associated with the onset of infections and respiratory disorders, the latter probably attributable to post-infusion reactions (Syed, 2018; Theriault and Solomon, 2020). In agreement with literature, Sicilian data showed a higher onset of PML with NTZ and DMF (Boffa et al., 2020), confirmed by antibody positivity to the JC virus. Activated B cells which host the JC virus probably promote viral replication and neurotropism via genetic transformation with increased risk of PML (Major, 2010).

Some unexpected ADRs based on SmPCs of MS drugs were retrieved, for which ROR values were significant. Based on available scientific literature, some of these associations could require further investigations. Reports related to FNG, NTZ, and IFN β-1a had mostly ADRs involving metabolic disorders, especially dyslipidemia, even if a retrospective observational study suggests that routine lipid profile monitoring is unnecessary during FNG treatment in MS patients without pre-existing cardiovascular comorbidities (Rauma et al., 2020). A recent study has shown the correlation between MS-related systemic inflammation and the onset of mild dyslipidemia, especially in males in the early stages of the disease (Rádiková et al., 2020). Moreover, dyslipidemia can influence, as a cardiovascular risk factor, the course of the disease, leading to a greater risk of progression to disability (Kowalec et al., 2017). However, treatment with statins showed mixed evidence (Tettey et al., 2014); in particular, that statins do not appear to have an effect on IFN β-1a-induced dyslipidemia (Rudick et al., 2009). Another potential safety signal concerned spontaneous abortion in pregnant women treated with NTZ. Animal studies have shown reproductive toxicity, but clinical data from clinical trials, prospective pregnancy registry, post-marketing cases, and available literature do not suggest an effect of NTZ exposure on pregnancy outcomes (Rommer and Zettl, 2018).

Malignancies mainly involved cases of skin tumors, including malignant melanoma and basal cell carcinoma, and lymphoma as has been demonstrated in some studies (Carbone et al., 2020; Lebrun-Frenay et al., 2020). The other types of tumors were unexpected. The risk of cancer associated with MS therapies has been debated by the scientific community, especially with FNG, NTZ, and other immunomodulatory drugs (Lebrun and Rocher, 2018). No difference in the incidence rates of the type of malignancies were observed between patients treated with NTZ and those treated with placebo. However, an effect of NTZ on malignant tumors cannot be ruled out. It is conceivable that the α4β1 integrin block could interfere with the activation of antigen-specific T lymphocytes and their ability to migrate to tumor sites (Förster et al., 2019). Moreover, in a post-marketing study, DMF, NTZ, and FNG were significantly related to a greater reporting of cancer (Dolladille et al., 2021). In this regard, only DMF had a positive association with the onset of breast cancer (Dolladille et al., 2021). However, DMF, especially at high concentrations, blocks nuclear factor κB (NFκB) activity in breast cancer, indicating an anticancer activity (Kastrati et al., 2016; Saidu et al., 2019). It has recently been noted that vitamin D deficiency is associated with an increased risk of MS (Sintzel et al., 2018), but there is no data to support immunostimulants, FNG, and TRF-induced hypovitaminosis. Concerning thyroid disorders, a recent review showed that approximately 6.44% of MS patients developed autoimmune thyroid disease, making it one of the most common comorbidity in MS, especially in patients treated with Alem (Marrie et al., 2015; Faissner and Gold, 2018).

### Limits and Strengths

This study is the first overview of ADRs related to the drugs approved for the treatment of MS based on a SRS database. SRS is the most used method to carry out post-marketing surveillance and to research ADRs not occurring during the preclinical and clinical studies (Barbieri et al., 2021a). Indeed, the post-marketing phase allows to analyze the onset of ADRs related to the use of DMTs in a real-world context, considering all risk factors probably associated with each individual event, such as comorbidities and polytherapy. The analysis of ROR does not allow to quantify the true risk of potential safety signals, especially for the limited number of cases; it only recommends a statistically significant disproportionality of detailed drug–ADR pairs, which should be further examined for signal validation (Raschi et al., 2019). Therefore, it is of primary importance that the identification of new and unexpected ADRs, which arise during the use of DMTs in patients affected by MS, help the clinician choose the best treatment. Nevertheless, this study has some limitations: the existence of under- or over-reporting of suspected ADRs and missing data are typical problems of SRS database; comorbidities, exam values, information on treatment and diagnostic tests are not always registered (Palleria et al., 2013). Furthermore, the lack of the total number of drug-exposed patients make it difficult to establish any meaningful conclusion around head to-head comparison and about the incidence of MS therapy-related ADRs (Pal et al., 2013). Moreover, a detailed description of ADR reports is available only for regional data reported into the Italian SRS database and related to Sicily. The aggregated data in the RAM system prevent the calculation of ROR at national level (Barbieri et al., 2021b). However, SRS helps to increase the knowledge of safety profiles in order to prevent some ADRs related to the use of drugs used in the treatment of MS, especially for those recently approved.

## Conclusion

The reporting of ADRs recorded for drugs used for the treatment of MS in Sicily has been relevant in recent years, thanks to some regional observational studies. Most of the ADRs highlighted in this study were already reported in the literature, but some unknown ADRs were also found. Disproportionality analysis showed potential risk of malignancies for FNG, NTZ, and DMF; moreover, FNG, NTZ, and IFN β-1a were mostly reported for dyslipidemia while abortion was unexpected in pregnant women treated with NTZ. These potential signals are not easy to evaluate, given the many factors that could be associated with their onset. Considering the rare and long-term ADRs that may arise in patients chronically treated for MS, pharmacovigilance activities are essential. However, further studies are necessary to increase the awareness about the safety profiles of new drugs entering the market, such as OCZ and Cladr, which currently have a current lower number of reports.

## Data Availability

The datasets generated for this study will not be made publicly available. National dataset in aggregated form is available online, while the access to the regional in single, non-aggregated dataset requires the approval of the Italian Medicines Agency, Further inquiries can be directed to MB, mbarbieri@unime.it.
