# Supplementary material for: Adverse Drug Reactions with Drugs Used in Multiple Sclerosis: An Analysis from the Italian Pharmacovigilance Database
Source: Front Pharmacol. 2022 Feb 23;13:808370. doi: 10.3389/fphar.2022.808370 (PMC8904918; doi:10.3389/fphar.2022.808370)
Supplement: Supplementary file 1 [file Table1.DOCX]

**Supplementary Table 1.** ADR grouping s by clustering the synonymous PTs of the same clinical condition under one term

| Clinical condition | Preferred Term (PT) |
| --- | --- |
| Ocular hyperaemia | Conjunctival hyperaemia |
|  | Ocular hyperaemia |
| Administration site hypersensitivity | Application site hypersensitivity |
|  | Injection site hypersensitivity |
|  | Administration site hypersensitivity |
| Administration site inflammation | Injection site inflammation |
|  | Administration site inflammation |
| Respiratory tract inflammation | Respiratory tract inflammation |
|  | Upper respiratory tract inflammation |
|  | Tracheal inflammation |
|  | Pharyngeal inflammation |
| Injection site induration | Puncture site induration |
|  | Injection site induration |
| Thyroid stimulating immunoglobulin increased | Thyroid stimulating immunoglobulin |
|  | Thyroid stimulating immunoglobulin increased |
| Monoclonal immunoglobulin increased | Monoclonal immunoglobulin increased |
|  | Monoclonal immunoglobulin present |
| Infusion site joint swelling | Application site joint swelling |
|  | Infusion site joint swelling |
| Lower limb fracture | Lower limb fracture |
|  | Ankle fracture |
|  | Femur fracture |
|  | Tibia fracture |
| Upper limb fracture | Wrist fracture |
|  | Radius fracture |
|  | Upper limb fracture |
|  | Hand fracture |
| Abnormal faeces | Abnormal faeces |
|  | Faeces hard |
|  | Faeces pale |
| Cardiac disorder | Cardiac discomfort |
|  | Cardiac disorder |
| Rash vesicular | Eczema vesicular |
|  | Rash vesicular |
| Application site erosion | Application site erosion |
|  | Injection site erosion |
| Genital erythema | Penile erythema |
|  | Genital erythema |
|  | Vulvovaginal erythema |
| Administration site joint erythema | Infusion site joint erythema |
|  | Injection site joint erythema |
|  | Administration site joint erythema |
| Eye haemorrhage | Eye haemorrhage |
|  | Conjunctival haemorrhage |
|  | Retinal haemorrhage |
| Injection site haemorrhage | Application site haemorrhage |
|  | Injection site haemorrhage |
| Gastrointestinal haemorrhage | Gastric haemorrhage |
|  | Gastrointestinal haemorrhage |
|  | Rectal haemorrhage |
| Injection site haematoma | Application site haematoma |
|  | Infusion site haematoma |
|  | Injection site haematoma |
| Administration site oedema | Application site oedema |
|  | Infusion site oedema |
|  | Injection site oedema |
|  | Administration site oedema |
| Administration site joint pain | Application site joint pain |
|  | Infusion site joint pain |
|  | Injection site joint pain |
|  | Administration site joint pain |
| Gastrointestinal disorder | Gastrointestinal disorder |
|  | Functional gastrointestinal disorder |
|  | Epigastric discomfort |
|  | Abdominal discomfort |
|  | Gastrointestinal motility disorder |
| Liver injury | Liver injury |
|  | Drug-induced liver injury |
|  | Hepatotoxicity |
|  | Liver disorder |
|  | Hepatic function abnormal |
| Partial seizures | Simple partial seizures |
|  | Partial seizures |
| COVID-19 | COVID-19 |
|  | Asymptomatic COVID-19 |
|  | Suspected COVID-19 |
|  | COVID-19 pneumonia |
|  | SARS-CoV-2 test positive |
| Lymphocytosis | Lymphocyte count increased |
|  | B-lymphocyte count increased |
|  | Lymphocytosis |
| Lymphopenia | Lymphocyte count decreased |
|  | B-lymphocyte count decreased |
|  | T-lymphocyte count decreased |
|  | CD4 lymphocytes decreased |
|  | CD8 lymphocytes decreased |
|  | Lymphopenia |
| Eosinophilia | Eosinophil count increased |
|  | Eosinophilia |
| Thrombocytopenia | Platelet count decreased |
|  | Thrombocytopenia |
| Neutropenia | Neutrophil count decreased |
|  | Neutropenia |
| Neutrophilia | Neutrophilia |
|  | Neutrophil count increased |
| Monocytosis | Monocyte count increased |
|  | Monocytosis |
| Monocytopenia | Monocyte count decreased |
|  | Monocytopenia |
| Leukopenia | Leukopenia |
|  | White blood cell count decreased |
| Leukocytosis | White blood cell count increased |
|  | Leukocytosis |
| Nasal congestion | Nasal congestion |
|  | Rebound nasal congestion |
| Asocial behaviour | Asocial behaviour |
|  | Social avoidant behaviour |
| Cystitis bacterial | Cystitis bacterial |
|  | Cystitis escherichia |
|  | Cystitis klebsiella |
|  | Cystitis pseudomonal |
| Injection site cellulitis | Application site cellulitis |
|  | Injection site cellulitis |
| Cervix carcinoma | Cervix carcinoma |
|  | Cervix carcinoma stage 0 |
|  | Cervix carcinoma stage III |
|  | Squamous cell carcinoma of the cervix |
|  | Adenosquamous carcinoma of the cervix |
|  | Adenocarcinoma of the cervix |
| Renal cancer | Renal cancer |
|  | Renal cancer stage I |
|  | Clear cell renal cell carcinoma |
|  | Renal cell carcinoma |
| Gastric cancer | Gastric cancer |
|  | Metastatic gastric cancer |
|  | Adenocarcinoma gastric |
| Ovarian cancer | Ovarian epithelial cancer |
|  | Ovarian cancer |
|  | Ovarian cancer metastatic |
|  | Ovarian cancer stage I |
| Oesophageal cancer | Oesophageal squamous cell carcinoma |
|  | Oesophageal carcinoma |
| Bladder cancer | Bladder cancer |
|  | Bladder cancer stage IV |
| Breast cancer | Breast cancer |
|  | Breast cancer female |
|  | Breast cancer metastatic |
|  | Breast cancer stage II |
|  | Invasive ductal breast carcinoma |
|  | Invasive breast carcinoma |
|  | Invasive lobular breast carcinoma |
|  | Intraductal proliferative breast lesion |
|  | Tubular breast carcinoma |
|  | Breast cancer in situ |
| Colon cancer | Colon cancer |
|  | Colon cancer metastatic |
|  | Adenocarcinoma of colon |
|  | Colorectal adenocarcinoma |
|  | Colorectal cancer |
| Thyroid cancer | Thyroid cancer |
|  | Papillary thyroid cancer |
| Pancreatic cancer | Adenocarcinoma pancreas |
|  | Ductal adenocarcinoma of pancreas |
|  | Intraductal papillary-mucinous carcinoma of pancreas |
|  | Pancreatic carcinoma |
| Injection site warmth | Infusion site warmth |
|  | Injection site warmth |
| Bacteriuria | Bacteriuria |
|  | Asymptomatic bacteriuria |
| Anti-thyroid antibody positive | Anti-thyroid antibody |
|  | Anti-thyroid antibody positive |
| Drug specific antibody present | Drug specific antibody |
|  | Drug specific antibody present |
| Antineutrophil cytoplasmic antibody increased | Antineutrophil cytoplasmic antibody increased |
|  | Antineutrophil cytoplasmic antibody positive |
| Anxiety | Anxiety |
|  | Anticipatory anxiety |
|  | Anxiety disorder |
|  | Social anxiety disorder |
| Administration site discolouration | Application site discolouration |
|  | Injection site discolouration |
|  | Administration site discolouration |
| Abortion | Abortion |
|  | Abortion induced |
|  | Abortion early |
|  | Abortion spontaneous |
| Hypoalbuminaemia | Hypoalbuminaemia |
|  | Blood albumin decreased |
| Hyperalbuminaemia | Hyperalbuminaemia |
|  | Blood albumin increased |
| Lung cancer | Lung adenocarcinoma |
|  | Lung adenocarcinoma stage IV |
|  | Small cell lung cancer |
|  | Lung cancer metastatic |
|  | Lung squamous cell carcinoma metastatic |
|  | Lung neoplasm malignant |
| Vomiting | Vomiting |
|  | Retching |
| Alopecia | Alopecia |
|  | Alopecia areata |
|  | Diffuse alopecia |
|  | Alopecia totalis |
|  | Alopecia universalis |
| Hypertransaminasemia | Transaminases increased |
|  | Hypertransaminasemia |
| Pyrexia | Post procedural fever |
|  | Rheumatic fever |
|  | Body temperature increased |
|  | Pyrexia |
|  | Body temperature fluctuation |
|  | Q fever |
|  | Relapsing fever |
| Rash | Skin reaction |
|  | Skin lesion |
|  | Rash |
|  | Rash erythematous |
|  | Erythema |
|  | Drug eruption |
|  | Skin irritation |
|  | Skin exfoliation |
|  | Toxic skin eruption |
|  | Eczema |
|  | Fixed eruption |
| Administration site rash | Application site erythema |
|  | Infusion site erythema |
|  | Injection site erythema |
|  | Instillation site erythema |
|  | Puncture site erythema |
|  | Administration site erythema |
|  | Injection site rash |
|  | Administration site rash |
|  | Injection site exfoliation |
| Burning sensation | Burning sensation |
|  | Skin burning sensation |
|  | Genital burning sensation |
| Troponin increased | Troponin increased |
|  | Troponin I increased |
| Administration site pain | Infusion site pain |
|  | Injection site pain |
|  | Administration site pain |
|  | Implant site pain |
|  | Puncture site pain |
| Abdominal pain | Abdominal pain |
|  | Abdominal tenderness |
|  | Abdominal pain lower |
|  | Abdominal pain upper |
|  | Gastrointestinal pain |
| Chest pain | Chest pain |
|  | Musculoskeletal chest pain |
|  | Non-cardiac chest pain |
| Oedema | Oedema |
|  | Generalized oedema |
| Bradycardia | Heart rate decreased |
|  | Sinus bradycardia |
|  | Bradycardia |
|  | Bradyarrhythmia |
| Cardiac fibrillation | Atrial fibrillation |
|  | Cardiac fibrillation |
|  | Ventricular fibrillation |
| Tachycardia | Sinus tachycardia |
|  | Supraventricular tachycardia |
|  | Ventricular tachycardia |
|  | Heart rate increased |
|  | Cardiac flutter |
| Arrhythmia | Arrhythmia |
|  | Sinus arrhythmia |
|  | Ventricular arrhythmia |
| Hypersensitivity | Hypersensitivity |
|  | Drug hypersensitivity |
|  | Type I hypersensitivity |
| Hypertension | Hypertension |
|  | Blood pressure increased |
|  | Blood pressure systolic increased |
|  | Hypertensive crisis |
|  | Diastolic hypertension |
|  | Gestational hypertension |
|  | Labile hypertension |
|  | Malignant hypertension |
|  | Systolic hypertension |
| Hyposideraemia | Hyposideraemia |
|  | Blood iron decreased |
| Neuropathy peripheral | Neuropathy peripheral |
|  | Acute motor-sensory axonal neuropathy |
|  | Peripheral sensory neuropathy |
| Hypotension | Blood pressure decreased |
|  | Hypotension |
|  | Diastolic hypotension |
|  | Orthostatic hypotension |
| Hyperbilirubinaemia | Blood bilirubin increased |
|  | Hyperbilirubinaemia |
| Aerophagia | Aerophagia |
|  | Flatulence |
| Drug ineffective | Drug ineffective |
|  | Therapy non-responder |
| Cough | Cough |
|  | Productive cough |
| Asthenia | Asthenia |
|  | Fatigue |
| Hot flushes | Feeling hot |
|  | Hot flushes |
| Dyspnea | Dyspnea |
|  | Dyspnea at rest |
|  | Dyspnea exertional |
|  | Nocturnal dyspnea |
|  | Respiratory fatigue |
| Choking | Choking sensation |
|  | Suffocation feeling |
|  | Sense of oppression |
|  | Choking |
| Anaemia | Anaemia |
|  | Iron deficiency anaemia |
|  | Hemolytic anaemia |
|  | Autoimmune haemolytic anaemia |
|  | Coombs positive haemolytic anaemia |
|  | Warm type haemolytic anaemia |
|  | Hypochromic anaemia |
|  | Microcytic anaemia |
|  | Thalassaemia minor |
| Oral candidiasis | Oral candidiasis |
|  | Oropharyngeal candidiasis |
| Hypercreatininaemia | Blood creatinine increased |
|  | Hypercreatininaemia |
| Visual impairment | Visual impairment |
|  | Visual field defect |
| Oral pain | Oral pain |
|  | Oropharyngeal pain |
| Eye oedema | Eyelid oedema |
|  | Cystoid macular oedema |
|  | Eye oedema |
|  | Periorbital oedema |
|  | Macular oedema |
| Extrasystoles | Extrasystoles |
|  | Supraventricular extrasystoles |
|  | Ventricular extrasystoles |
| Gastroenteritis | Gastritis |
|  | Gastroenteritis |
|  | Enteritis |
| Ocular hyperaemia | Conjunctival hyperemia |
|  | Ocular hyperaemia |
| Skin wound | Skin wound |
|  | Skin abrasion |
|  | Skin lesion |
|  | Skin injury |
| Mood swings | Mood altered |
|  | Mood swings |
| Onycholysis | Onycholysis |
|  | Onychomadesis |
| Rhinitis | Rhinitis |
|  | Rhinitis allergic |
| Administration site extravasation | Infusion site extravasation |
|  | Injection site extravasation |
| Off-label use | Product use in unapproved indication |
|  | Off-label use |
| Urinary tract infection | Urinary tract infection |
|  | Urinary tract infection bacterial |
|  | Urinary tract infection fungal |
|  | Escherichia urinary tract infection |
|  | Urinary tract infection enterococcal |
| Varicella zoster virus infection | Varicella zoster virus infection |
|  | Disseminated varicella-zoster infection |
| Administration site infection | Injection site infection |
|  | Administration site infection |
| Viral infection | Cytomegalovirus infection |
|  | Viral infection |
|  | Coronavirus infection |
|  | Herpes virus infection |
|  | Papilloma viral infection |
|  | Parvovirus B19 infection |
|  | Human polyomavirus infection |
|  | Epstein-Barr virus infection |
|  | Parainfluenzae virus infection |
|  | Molluscum contagiosum |
|  | Infectious mononucleosis |
|  | Measles |
|  | Mononucleosis syndrome |
| Bacterial infection | Chlamydial infection |
|  | Escherichia infection |
|  | Bacterial infection |
|  | Helicobacter infection |
|  | Klebsiella infection |
|  | Morganella infection |
|  | Mycoplasma infection |
|  | Proteus infection |
|  | Ureaplasma infection |
|  | Enterococcal infection |
|  | Pneumococcal infection |
|  | Staphylococcal infection |
|  | Streptococcal infection |
|  | Rickettsiosis |
|  | Syphilis |
| Insomnia | Insomnia |
|  | Initial insomnia |
|  | Middle insomnia |
|  | Poor quality sleep |
| Hepatic failure | Hepatic failure |
|  | Acute hepatic failure |
| Respiratory failure | Respiratory failure |
|  | Acute respiratory failure |
| Dyslipidaemia | Dyslipidaemia |
|  | Hyperlipidaemia |
|  | Hypercholesterolaemia |
|  | Hypertriglyceridaemia |
|  | Blood triglycerides increased |
|  | Blood cholesterol increased |
| Hyperthyroidism | Hyperthyroidism |
|  | Primary hyperthyroidism |
|  | Secondary hyperthyroidism |
| Application site hypertrophy | Application site hypertrophy |
|  | Injection site hypertrophy |
| Hypothyroidism | Hypothyroidism |
|  | Autoimmune hypothyroidism |
|  | Post procedural hypothyroidism |
|  | Primary hypothyroidism |
| Administration site irritation | Injection site irritation |
|  | Administration site irritation |
| Leiomyosarcoma | Leiomyosarcoma |
|  | Pleomorphic leiomyosarcoma |
| Administration site injury | Infusion site injury |
|  | Injection site injury |
| Lipodystrophy | Lipodystrophy acquired |
|  | Partial lipodystrophy |
| Administration site bruise | Application site bruise |
|  | Administration site bruise |
| Malignant melanoma | Superficial spreading melanoma stage III |
|  | Metastatic malignant melanoma |
|  | Superficial spreading melanoma stage IV |
|  | Superficial spreading melanoma stage unspecified |
|  | Choroid melanoma |
|  | Malignant melanoma |
|  | Malignant melanoma in situ |
|  | Naevoid melanoma |
|  | Melanoma recurrent |
|  | Nodular melanoma |
| Meningitis bacterial | Meningitis bacterial |
|  | Meningitis listeria |
|  | Meningitis pneumococcal |
|  | Meningitis staphylococcal |
| Myocarditis | Myocarditis |
|  | Autoimmune myocarditis |
|  | Viral myocarditis |
| Death | Death |
|  | Sudden death |
| Administration site necrosis | Application site necrosis |
|  | Injection site necrosis |
| Naevus | Dysplastic naevus |
|  | Epidermal naevus |
|  | Melanocytic naevus |
|  | Congenital melanocytic naevus |
|  | Eye naevus |
| Administration site nodule | Application site nodule |
|  | Infusion site nodule |
|  | Injection site nodule |
|  | Administration site nodule |
| Urticaria | Urticaria |
|  | Urticaria chronic |
|  | Mechanical urticaria |
| Application site urticaria | Application site urticaria |
|  | Infusion site urticaria |
|  | Injection site urticaria |
| Otitis media | Otitis media |
|  | Otitis media acute |
| Duodenal Perforation | Duodenal ulcer perforation |
|  | Duodenal perforation |
| Pericarditis | Pericarditis |
|  | Viral pericarditis |
| Pyelonephritis | Pyelonephritis |
|  | Pyelonephritis acute |
| Pneumothorax | Pneumothorax |
|  | Pneumothorax spontaneous |
| Polyneuropathy | Polyneuropathy |
|  | Demyelinating polyneuropathy |
| Pneumonia bacterial | Pneumonia bacterial |
|  | Pneumonia haemophilus |
|  | Pneumonia legionella |
|  | Pneumonia mycoplasmal |
|  | Pneumonia staphylococcal |
| Pneumonia viral | Pneumonia viral |
|  | Pneumonia cytomegaloviral |
|  | Pneumonia influenzal |
| Hypokalaemia | Hypokalaemia |
|  | Blood potassium decreased |
| Administration site pruritus | Application site pruritus |
|  | Infusion site pruritus |
|  | Injection site pruritus |
|  | Instillation site pruritus |
|  | Administration site pruritus |
| Administration site pustule | Injection site pustule |
|  | Administration site pustule |
| Administration related reaction | Administration related reaction |
|  | Infusion related reaction |
|  | Injection related reaction |
|  | Immediate post-injection reaction |
|  | Infusion site reaction |
|  | Injection site reaction |
|  | Puncture site reaction |
|  | Administration site reaction |
| Infection reactivation | Hepatitis B reactivation |
|  | Herpes zoster reactivation |
|  | Infection reactivation |
|  | Cytomegalovirus infection reactivation |
|  | Epstein-Barr virus infection reactivation |
| Musculoskeletal stiffness | Muscle rigidity |
|  | Musculoskeletal stiffness |
| Testicular cancer | Testicular seminoma (pure) |
|  | Testicular seminoma (pure) stage I |
|  | Extragonadal primary seminoma (pure) stage I |
|  | Testicular malignant teratoma |
| Bacterial sepsis | Escherichia sepsis |
|  | Listeria sepsis |
|  | Bacterial sepsis |
|  | Staphylococcal sepsis |
| Underdose | Underdose |
|  | Accidental underdose |
|  | Intentional underdose |
|  | Prescribed underdose |
| Overdose | Overdose |
|  | Intentional overdose |
|  | Prescribed overdose |
| Cholecystitis | Cholecystitis |
|  | Cholecystitis acute |
|  | Cholecystitis chronic |
|  | Cholecystitis infective |
| Hallucination | Hallucination |
|  | Hallucination, auditory |
|  | Hallucination, visual |
|  | Hallucinations, mixed |
| Amylase increased | Amylase |
|  | Amylase increased |
| Angina pectoris | Angina pectoris |
|  | Angina unstable |
| Genital herpes | Genital herpes |
|  | Genital herpes simplex |
|  | Genital herpes zoster |
| Herpes ophthalmic | Herpes ophthalmic |
|  | Ophthalmic herpes simplex |
|  | Ophthalmic herpes zoster |
| Thrombocytosis | Thrombocytosis |
|  | Platelet count increased |
| Pharyngitis bacterial | Pharyngitis bacterial |
|  | Staphylococcal pharyngitis |
| Multiple sclerosis relapse | Multiple sclerosis relapse |
|  | Multiple sclerosis |
|  | Primary progressive multiple sclerosis |
|  | Progressive multiple sclerosis |
|  | Relapsing multiple sclerosis |
|  | Relapsing-remitting multiple sclerosis |
|  | Secondary progressive multiple sclerosis |
|  | Condition aggravated |
|  | Disease progression |
|  | Disease recurrence |
| Headache | Cluster headache |
|  | Exertional headache |
|  | Headache |
|  | Tension headache |
|  | Migraine |
|  | Migraine with aura |
|  | Migraine without aura |
